# Successful removal of a complicated hydatid cyst via bronchoscopy and without surgery: A rare case report

**DOI:** 10.1016/j.ijscr.2025.111675

**Published:** 2025-07-14

**Authors:** Amir M.H. Asnaashari, Zahra Behrooznia, Amir Hossein Jafarian, Mohammad Javad Najafzadeh, Amir Baniasad

**Affiliations:** a Lung Disease Research Center, Mashhad University of Medical Sciences, Mashhad, Iran; b Cancer and Molecular Research Center, Department of Pathology, Ghaem Hospital, School of Medicine, Mashhad University of Medical Sciences, Mashhad, Iran; c Faculty of Medicine, Kerman University of Medical Sciences, Kerman, Iran

**Keywords:** Echinococcosis, Hydatid cyst, Bronchoscopy, Pneumonectomy

## Abstract

**Introduction:**

Pulmonary hydatid cysts have always been associated with diagnostic and therapeutic challenges, especially in endemic areas. While surgical resection is the primary treatment for large cysts, bronchoscopy has been used as a potential alternative for selected cases.

**Presentation of case:**

We report a rare case of a 70-year-old diabetic patient with a complex pulmonary hydatid cyst who was successfully managed by bronchoscopy without surgical intervention. The patient presented with a nonproductive cough, and imaging showed a heterogeneous non-cavitated mass with central necrosis. During diagnostic bronchoscopy, cystic components were identified and removed, and histopathological evaluations confirmed the hydatid cyst.

**Discussion:**

The patient was treated with albendazole and showed significant improvement in imaging after one month.

**Conclusion:**

This case highlights the potential of bronchoscopy as a minimally invasive diagnostic and therapeutic tool for pulmonary hydatid disease, reducing the need for surgery in high-risk patients.

## Highlights

• Rare hydatid cyst removed via bronchoscopy without surgical intervention.
• Bronchoscopy enabled direct visualization and extraction of cystic material.
• Diagnosis confirmed by histopathology and treated with albendazole.
• Patient improved without need for high-risk surgery.
• Bronchoscopy offers a safe option for complex cysts in high-risk patients.

## 1 Introduction

A serious zoonotic health risk is echinococcosis or hydatidosis, especially in areas where pastoral activities are endemic. It has a financial and public health burden. Human echinococcosis is a neglected zoonosis caused by parasites of the genus *Echinococcus,* and it causes severe morbidity and disability [1,2]. The lung (20 %–30 %) is the second most frequently affected organ by hydatid cysts after the liver (60 %–80 %) [3]. Small hydatid cysts often cause no symptoms, while large cysts usually cause chest pain, cough, shortness of breath, and hemoptysis [4].

A hydatid cyst's size, location, and symptoms determine the medical and/or surgical intervention needed to treat it. The most favored form of treatment is surgical intervention (lobectomy or cystectomy) [5]. Complete removal of a pulmonary hydatid cyst via bronchoscopy is rare, as bronchoscopy is not routinely performed to diagnose them [6]. Surgery remains the treatment for large cysts, but medical management may be considered for small cysts. Although rare, successful extraction of hydatid cysts via bronchoscopy without surgical resection has been documented [7,8]. Flexible bronchoscopy should be considered in pulmonary hydatid cysts to reduce the complications associated with open surgery.

The use of bronchoscopy in diagnosing and treating lung cyst hydatid remains controversial [9]. Our case report presents a 70-year-old diabetic patient with a complicated pulmonary hydatid cyst, which was successfully managed through bronchoscopy. It underscores this technique's diagnostic and therapeutic utility in managing pulmonary hydatid cysts. Our case has been reported in line with the SCARE criteria [10].

## 2 Case description

A 70-year-old farmer man with a history of diabetes was admitted to our tertiary hospital with the chief complaint of non-productive coughing from 40 days ago. The coughing was repeated several times daily, and their frequency was progressive. Sometimes, coughing was associated with a small amount of non-purulent sputum. The patient had anorexia and weight loss during the last two months (about 10 kg). The patient had no history of hemoptysis, dyspnea, fever, or chest pain.

The patient had the history of hypertension, hyperlipidemia, and diabetes from more than ten years ago. He had no history of addiction or smoking. He consumed metformin (500 mg twice daily), sitagliptin (50 mg once daily), losartan (25 mg twice daily), and atorvastatin (40 mg once daily) a long time ago. The patient had no family history of similar symptoms and diseases in his first-grade relatives. The patient's previous job was farming and herding.

The patient was fully awake during a physical examination and had no respiratory distress. The respiratory rate was 18/min, blood pressure was 130/70 mmHg, pulse rate was 91/min, and axillary temperature was 37 °C. The left lung sounds in lower zones were decreased in auscultation. His Body Mass Index (BMI) was calculated at 27 kg/m^2^.

## 3 Diagnostic assessment

The patient underwent a lung computed tomography (CT) scan. Fig. A1 shows a heterogeneous mass with dimensions of 80*60 mm was observed in the left lower lobe. The central zones of the mass were necrotic. As shown in Table 1, laboratory tests were requested for the patient, which was compatible with poor control diabetes.

**Fig. A:**
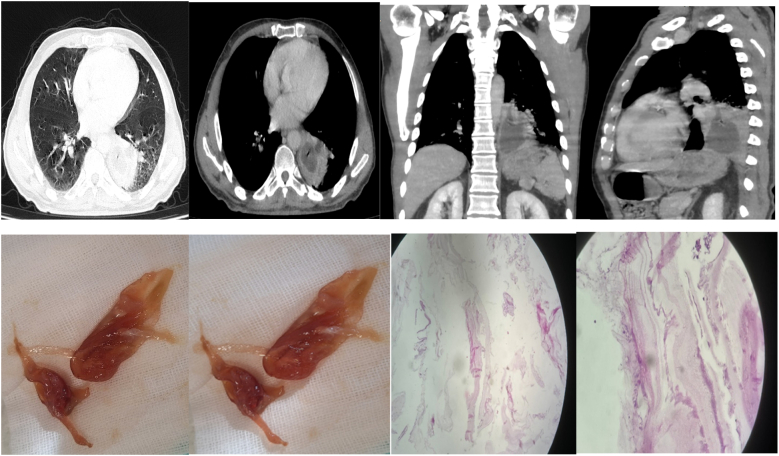
1. Lung CT scan of the patient with a heterogeneous mass with the dimensions of 80*60 mm in the left lower lobe. Fig. A2. The two about 50*30 and 20*30 mm membranous structures were extracted after suctioning the left lower lobe bronchus during bronchoscopy. Fig. A3. Lamella wall of hydatid cyst and mucoid secretions, confirming the diagnosis of hydatid cyst in histopathologic assessment.

**Table 1:** Primary laboratory findings of the patient.

Investigations	Results	Reference range
CRP (mg/dL)	3.3	0–6
WBC (×103/uL)	6.68	4–10
RBC (×106/uL)	5.23	4.5–5.5
Hb (gr/dL)	14.4	13–17
Hct (%)	44.4	40–50
Platelet (×109/uL)	396	150–450
Absolute neutrophil count (×103/uL)	3.39	–
Lymphocyte (×103/uL)	2.76	–
FBG (mg/dL)	129	70–100
Hemoglobin A1c (%)	11.2	≤ 5.6

CRP, C-reactive protein; ESR, Erythrocyte sedimentation rate; WBC, White blood cells; RBC, Red blood cells; Hb, Hemoglobin; Hct, Hematocrit; FBG, Fasting blood glucose.

The patient underwent a bronchoscopy with general anesthesia (midazolam and propofol, along with topical lidocaine applied to the laryngeal area). During bronchoscopy, an initially observed large clot obstructing the orifice of the left lower lobe bronchus was carefully extracted using suction and lavage alone through a flexible bronchoscope, without the need for forceps, snares, or rigid instrumentation. Upon removal, the specimen exhibited a membranous-like appearance on gross examination. Two fragments, measuring approximately 50 × 30 mm and 20 × 30 mm, were retrieved and submitted for histopathological analysis (Fig. A2). Many purulent secretions were extracted after removing the membrane, which was sent concurrently with bronchoalveolar lavage (BAL) to assess acid-fast bacillus (AFB), microorganism culture, and cytology. Due to the equipment limitations, no high-resolution bronchoscopic images were captured during the intervention. At the time of the procedure, we did not anticipate encountering such a lesion and therefore had no prior intention to extract it. Consequently, the procedure was planned with a flexible bronchoscope, which was readily available. Additionally, the location of the lesion made it accessible only through flexible bronchoscopy. While rigid bronchoscopy provides greater airway control and is preferred in complex resections, it was not available in our center.

The results of the BAL assessment showed no evidence of AFB in Ziehl-Neelsen staining. The BAL fluid cytology showed bronchial and squamous cells and alveolar macrophages mixed with many polymorphonuclear cells compatible with acute inflammatory fluid. The histopathology assessment of the membranous-like lesion showed the lamella wall of a hydatid cyst and mucoid secretions suggestive of a hydatid cyst. Hematoxylin and Eosin Stain (H&E) x100 show mucoid secretion and eosinophils lamellar wall of hydatid cyst and scattered inflammatory cells confirming the diagnosis of hydatid cyst (Fig. A3). Although serological tests such as ELISA are commonly used to support the diagnosis of hydatid disease, they were not performed in this case. The diagnosis was confirmed through direct visualization of cystic structures during bronchoscopy and subsequent histopathological confirmation. The availability of histologic tissue and radiologic findings made serology unnecessary for diagnosis in this patient.

### 3.1 Outcome and follow-up

The patient was admitted for two days. The day after the bronchoscopy, the patient's lung CT scan was repeated, and a cavitary lesion with peripheral consolidation was observed in the left lower lobe of the lung (Fig. B).

**Fig. B:**
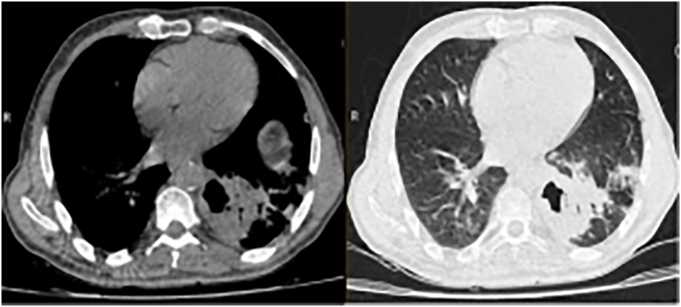
The cavitary lesion with peripheral consolidation in the left lower lobe of the lung after bronchoscopy and suctioning of the membranous.

The treatment with albendazole (400 mg twice daily), cefixime (400 mg once daily until two weeks), and clindamycin (300 mg four times daily) was prescribed for the patient. The patient was advised to have a follow-up visit after one month.

After four months, the patient underwent a lung CT scan, which showed a decrease in the size of the cavitary lesion and peripheral consolidation (Fig. C). The cough was diminished. The clindamycin was discontinued, but the treatment with albendazole continued for the patient.

**Fig. C:**
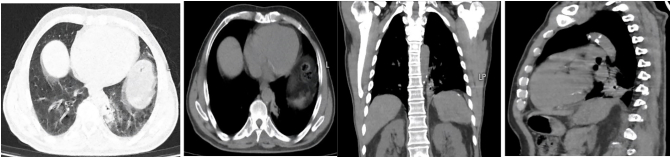
Decrease in size of cavitary lesion and peripheral consolidation after four months in lung CT scan.

## 4 Discussion

Despite being less prevalent than hepatic involvement, pulmonary hydatid disease presents diagnostic and treatment challenges because of its varied clinical presentation and potential for consequences such bronchial rupture or obstruction [11]. In this case, we observed nonspecific signs and symptoms, including nonproductive cough, anorexia, and weight loss, which delayed the definitive diagnosis until imaging or invasive procedures were performed. Commonly reported symptoms include cough, dyspnea, hemoptysis, and chest pain, usually following lung tissue rupture [12]. Furthermore, hydatid cysts can mimic the manifestations of other diseases such as cancer and tuberculosis, as in our case, weight loss and anorexia can be misleading. Cystic echinococcosis, or hydatid cyst disease, is a global public health concern common to rural communities. The disease is transmitted through the ingestion of contaminated vegetables, food, and water containing parasite eggs [6]. In our case, a history of farming and herding is a common risk factor for echinococcosis in endemic areas.

Combining imaging, serology, and epidemiological risk factors is the most effective method of diagnosing pulmonary hydatid cysts. Pulmonary hydatid cysts appear on CT as cystic lesions with smooth walls of variable thickness and a homogeneous internal fluid density content [[12], [13], [14]]. In this case, identifying a heterogeneous mass with cystic components on the initial lung CT scan prompted a differential diagnosis that included malignancy, abscess, or parasitic infection, emphasizing the importance of integrating radiographic findings with clinical history in endemic areas.

The presence of a yellowish to whitish gelatinous endobronchial membrane is the most distinctive bronchoscopy finding [13,15]. In our case, a similar membranous-like lesion was observed on bronchoscopy that obstructed the left lower lobe bronchus. On further histopathological evaluation, the membranous lesion was like the lamellar wall of a hydatid cyst, and the mucous discharge was suggestive of a hydatid cyst. Pulmonary hydatid cysts are treated medically or surgically based on size, location, and symptoms. Medical therapy with albendazole is recommended for small cysts (<5 cm) or patients with surgical contraindications. Surgery remains the primary curative approach, with precautions to prevent intraoperative cyst rupture and parasite dissemination [16,17].

Complete removal of pulmonary hydatid cysts via bronchoscopy is rare. It has been reported in a few cases, mainly as an unexpected diagnosis, as bronchoscopy is not routinely used to treat pulmonary hydatid cysts [7,8,[18], [19], [20]]. Attempting to remove cystic material in a confined space using a bronchoscope and saline may increase the risk of cyst rupture and lead to allergic reactions and even anaphylaxis [8,9].

Two 2024 case reports demonstrated that bronchoscopy can be an effective treatment for pulmonary hydatid cysts, especially in select patients. Wen et al. [6] and Karcıoğlu et al. [9] showed successful cyst removal via bronchoscopy in cases involving ruptured or non-surgically treated cysts. We present a more complex case involving an elderly, diabetic patient with an unruptured, necrotic cyst. Despite the higher risk, bronchoscopic removal was successful, with no complications or recurrence. This case is notable for its detailed clinical and radiologic documentation and supports the growing role of bronchoscopy as both a diagnostic and therapeutic option, especially for high-risk surgical patients.

Although bronchoscopy has been of great interest for the evaluation of suspected ruptured pulmonary hydatid cysts (due to prior cyst rupture and limited risk of anaphylaxis), our case suggests that bronchoscopy may be available as a minimally invasive alternative to surgery in selected patients with moderately sized hydatid cysts, especially when surgical risks are high, such as in this elderly patient with comorbidities such as diabetes.

Similar to our case, pulmonary hydatid cysts are mostly discovered incidentally and can even be detected during diagnostic endoscopy, increasing the risk of anaphylactic reactions. However, since the ruptured material may have been completely removed during diagnostic bronchoscopy in small to medium-sized cysts, we recommend that medical treatment be initiated and surgery be postponed. Given that the recurrence of hydatid cysts in other parts of the lung is very high, our approach will avoid early lung surgery.

In our case, a follow-up lung CT scan revealed a reduction in the size of the cavitary lesion along with peripheral consolidation. In addition, the patient's cough had improved. However, the rarity of our approach necessitates further studies to establish its safety, efficacy, and indications compared to traditional surgical methods.

## 5 Conclusion

This case highlights the utility of bronchoscopy in the simultaneous diagnosis and treatment of complex pulmonary hydatid cysts, especially in patients with high surgical risk. While surgery remains the standard treatment for large cysts, our findings suggest that during diagnostic bronchoscopy and incidental detection of small to medium-sized hydatid cysts, complete removal of the cystic lesions and conventional medical therapy may be a viable alternative in selected cases. Further studies are needed to demonstrate the safety and efficacy of this approach compared with conventional surgical methods.

## Consent statement

Written informed consent was obtained from the patient to publish this report in accordance with the journal's patient consent policy.

## Ethics statement

Written informed consent was obtained from the patient to publish this report in accordance with the journal's patient consent policy. This study was approved by the Ethics Committee of Mashhad University of Medical Sciences with code IR.MUMS.REC.1404.038.

## Guarantor

Dr. Amir Baniasasd

## Declaration of Generative AI and AI-assisted technologies in the writing process

The author(s) declare that no Gen AI was used in the creation of this manuscript.

## Funding statement

There is no external funding source for this case report.

## CRediT authorship contribution statement

**Amir M.H. Asnaashari:** Data curation, Validation, Writing – review & editing. **Zahra Behrooznia:** Conceptualization, Investigation, Resources, Writing – original draft. **Amir Hossein Jafarian:** Supervision, Visualization, Writing – original draft. **Mohammad Javad Najafzadeh:** Investigation, Writing – review & editing. **Amir Baniasad:** Validation, Writing – original draft.

## Declaration of competing interest

There is no conflict of interest.

## Data Availability

The data supporting this study's findings are available from the corresponding authors upon reasonable request.

## References

[1] Gessese A.T. (2020). Review on epidemiology and public health significance of hydatidosis. Veterinary Medicine International.

[2] Otero-Abad B., Torgerson P.R. (2013). A systematic review of the epidemiology of echinococcosis in domestic and wild animals. PLoS Negl. Trop. Dis..

[3] Aydin Y., Ulas A.B., Ahmed A.G., Eroglu A. (2022). Pulmonary hydatid cyst in children and adults: diagnosis and management. Eurasian J. Med..

[4] Goussard P., Eber E., Mfingwana L., Nel P., Schubert P., Janson J. (2022). Paediatric pulmonary echinococcosis: a neglected disease. Paediatr. Respir. Rev..

[5] Banihashemi S.H., Banihashemi S.H., Karimi A., Yousefabad S.H.A., Askari Y., Aghajanzadeh M. (2024). Investigating pulmonary hydatidosis: a narrative review. Hormozgan Med. J..

[6] Wen K.Z., Lim R.T., Dimitri A., Noonan L., Williamson J. (2024). Complete removal of a ruptured pulmonary hydatid cyst during conscious sedation bronchoscopy: a case report and literature review. Respirol. Case Rep..

[7] Hejazi M.E., Tekantapeh S.T., Hasani S. (2018). A novel bronchoscope method (saline injection method) for complete extraction of ruptured pulmonary hydatid cyst. Clin. Respir. J..

[8] Zamani A., Yosunkaya S. (2018). Intact endobronchial hydatid cyst: an unexpected bronchoscopic challenge. Asian Cardiovascular and Thoracic Annals..

[9] Karcioglu O., Kara A., Kurtulan O., Uysal S., Selçuk Z.T. (2024). Lung cyst hydatid extracted via bronchoscopy and the necessity of surgery: a case report. Iran. J. Parasitol..

[10] Ahmed K., Ahmed A., Ginimol M., Catrin S., Rasha R., Thomas F. (2025). Revised Surgical CAse REport (SCARE) guideline: an update for the age of artificial intelligence. Premier J. Sci..

[11] Lupia T., Corcione S., Guerrera F., Costardi L., Ruffini E., Pinna S.M., Rosa F.G.D. (2021). Pulmonary echinococcosis or lung hydatidosis: a narrative review. Surg. Infect..

[12] Rawat S., Kumar R., Raja J., Singh R.S., Thingnam S.K.S. (2019). Pulmonary hydatid cyst: review of literature. J. Family Med. Prim. Care.

[13] Sarkar M., Pathania R., Jhobta A., Thakur B.R., Chopra R. (2016). Cystic pulmonary hydatidosis. Lung India.

[14] Fakhar M., Keighobadi M., Hezarjaribi H.Z., Montazeri M., Banimostafavi E.S., Sayyadi S. (2021). Two decades of echinococcosis/hydatidosis research: bibliometric analysis based on the web of science core collection databases (2000–2019). Food and Waterborne Parasitology.

[15] Mathew J.L., Kumar K., Singh M. (2021). Pulmonary hydatid cyst diagnosed by flexible fiberoptic bronchoscopy. J. Bronchol. Intervent. Pulmono..

[16] Brunetti E., Kern P., Vuitton D.A. (2010). Expert consensus for the diagnosis and treatment of cystic and alveolar echinococcosis in humans. Acta Trop..

[17] Mihetiu A., Bratu D., Neamtu B., Sabau D., Sandu A. (2024). Therapeutic options in hydatid hepatic cyst surgery: a retrospective analysis of three surgical approaches. Diagnostics.

[18] Alavi A., Aghajanzadeh M., Hejri G.M. (2010).

[19] Sharif A., Ansarin K., Rashidi F., Taghizadieh A. (2011). Bronchoscopic diagnosis and removal of a ruptured hydatid cyst. J. Bronchol. Intervent. Pulmono..

[20] Kunal S., Pilaniya V., Shah A. (2016). Middle lobe syndrome: a singularly rare presentation of complicated pulmonary hydatid disease. Case Rep. Dermatol..

